# The one-year prevalence of nonspecific back pain in public primary health care establishments among 1.7 million people in western Sweden

**DOI:** 10.1017/S1463423620000523

**Published:** 2020-11-20

**Authors:** Olof Thoreson, Anna Aminoff, Catharina Parai

**Affiliations:** 1 Department of Orthopedics, Institute of Clinical Sciences at Sahlgrenska Academy, University of Gothenburg, Gothenburg, Sweden; 2 Research and Development Primary Health Care Centre Gothenburg and Södra Bohuslän, Gothenburg, Sweden; 3 Wästerläkarna, Wästerläkarna AB, Sweden; 4 GHP Spine Center, Gothenburg, Sweden

**Keywords:** epidemiology, health behavior, musculo-skeletal conditions, primary health care, register

## Abstract

**Aim:**

The one-year prevalence of diagnosed nonspecific back pain in Sweden is not known. Thus, this observational register-based study aimed to evaluate this prevalence by using data from the Region Västra Götaland, inhabiting 1.7 million people.

**Methods:**

Data from 2014 to 2018 were extracted from the VEGA database register. This register holds all health data from the publicly funded health care establishments in Region Västra Götaland. Aggregated data are presented as the one-year prevalence of unique individuals diagnosed with nonspecific back pain (i.e., the ICD-10 code M54). Stratification by health care level, gender, age, and M54 sub-diagnoses were made.

**Findings:**

Between 2014 and 2018, the annual prevalence of diagnosed nonspecific back pain in public primary health care increased from 4.8% to 6.0% (26% increase, *P* < 0.001, CI 25–27%). In 2018, the one-year prevalence was 7.2% among women and 4.8% among men (50% difference, *P* = 0.001, CI 49–52%). The one-year prevalence increased by age, and the highest figure (11%) was seen in the age group of 80–84. Low back pain, M54.5, was the most common sub-diagnosis. The one-year prevalence was significantly higher (*P* < 0.001) among women in all the M54 sub-diagnoses.

**Conclusion:**

The one-year prevalence of diagnosed nonspecific back pain was 6% in public primary health care in 2018 and has increased since 2014. Women were diagnosed considerably more frequently than men. Publicly funded rehabilitation efforts, as well as actions focusing on the prevention of back pain, is probably money well spent.

## Introduction

Hardly anyone manages to go through life without a period of back pain, but how many seek medical care and get diagnosed with back pain?

Back pain is a common problem globally and the prevalence increases with age (Balague *et al.*, 1999; Meucci *et al.*, 2015). Despite methodological variation and definition differences between studies, reviews have displayed lifetime self-reported prevalence of back pain exceeding 80% (Balague *et al.*, 2012), 1-month prevalence of low back pain around 23% and point prevalence of low back pain of around 12% (Hoy *et al.*, 2012). Back pain is also considered to be the leading cause of activity limitation and work absence globally (Hoy *et al.*, 2014). A study based on primary health care medical records at 12 general practices in Britain in 2006 showed that the one-year consultation prevalence due to back pain was 5.9% (Jordan *et al.*, 2010). These numbers indicate that many people with back pain do not seek medical assistance or choose alternative medicine options. This was also supported by a study reporting that two-thirds of patients with low back pain did not consult with a general practitioner regarding their pain during the same year (Picavet *et al.*, 2008). Women appear to have a higher risk of both acute and chronic back pain (Hoy *et al.*, 2012; Maher *et al.*, 2017) and are more prone to seek medical consultations (Ferreira *et al.*, 2010; Wang *et al.*, 2013).

To establish effective prevention strategies and health care treatments, it is essential to determine the diagnosed one-year prevalence of nonspecific back pain in public health care in Sweden. Better prevention methods and treatments would reduce both personal disabilities and societal costs.

Back pain has traditionally been divided into different categories dependent on cause and symptoms (van Tulder *et al.*, 2006). Nonspecific back pain is the most common category of diagnoses and is used when the underlying cause is undetermined. Specific back pain is characterized by a known cause such as cancer and fractures, or disorders originating, for instance, from the abdomen or the hip. Back pain with neuralgia is usually a result of nerve root affection such as disc herniation or spinal stenosis.

In Sweden, public health care is divided into three levels: primary health care, specialized outpatient care, and inpatient care (Institute, 2019). The primary health care, consisting of general practitioners and physiotherapists, is usually the first health care provider that patients visit, but patients can also attend the hospital emergency departments and outpatient clinics without a referral. The public health care is funded mainly through taxes, and patient health care fees only cover a small percentage of the total cost. The health care providers are both private as well as public, and the same economic rules and structures apply for both. All health care visits and diagnoses are coded according to the WHO International Statistical Classification of Diseases and Related Health Problems – Tenth Revision (ICD-10) system.

Region Västra Götaland is one of 20 Swedish county councils responsible for financing public health care. A sixth of the Swedish population – 1.7 million – live in Region Västra Götaland, consisting of both rural and urban areas. The biggest city is Gothenburg with an inner-city population of around 560 000 (2015). Since 2014, all publicly funded health care in Västra Götaland report to the VEGA database register (Analytics, 2020). The health care economic compensation system in Region Västra Götaland is mainly derived from the reported variables for each health care clinic, and the care is analyzed according to the reported diagnoses of each clinic. Each diagnosis is linked to a specific health burden equation which is not public. The higher the burden – the higher the monetary compensation from the Region to the health care clinic. This economical arrangement is under governmental supervision through several control mechanisms of the data thatare reported to the VEGA register (Institute, 2019).

Benchmarking studies on the prevalence of diagnosed nonspecific back pain in Sweden have been lacking due to the absence of high-quality primary health care data. The VEGA register now provides the opportunity to fill this gap. As being important pillars in the distribution of funds and in health economic calculations, the reporting of accurate prevalence numbers is encouraged, (Nachemson and Jonsson, 2000; Maetzel and Li, 2002; Katz, 2006; Dagenais *et al.*, 2008; Wenig *et al.*, 2009). Thus, the aim was to evaluate the annual prevalence of nonspecific back pain, ICD-10 M54, in Region Västra Götaland, Sweden, from 2014 to 2018, and to examine differences between health care levels, gender, age, and M54 sub-classifications.

## Material and methods

In this observational register-based study, data from 2014 to 2018 were extracted from the VEGA register, with the whole population as the frame population. All patients diagnosed with an M54 code (i.e., nonspecific back pain) were included as the target population. In addition to the M54 variable, gender, age, the health care level in which the M54 code was diagnosed, number of health care establishments were withdrawn from the register. Aggregated data were presented as the one-year prevalence of unique individuals with any diagnosis of M54. Stratifications by health care level, gender, age, and M54 sub-classifications were made. The sub-classifications were M54.0 Panniculitis affecting regions of neck and back; M54.1 Radiculopathy; M54.2 Cervicalgia, M54.3 Sciatica, M54.4 Low back pain with sciatica; M54.5 Low back pain; M54.6 Pain in thoracic spine; M54.8 Other types of back pain; M54.9 back pain, unspecified.

All patients are uniquely sorted in the VEGA register through their individual identity number. Throughout a year, the same patient may receive a diagnosis from more than one of the health-care levels (primary, specialized inpatient, and outpatient care). However, in the VEGA register, the health care levels are separated from each other.

The one-year prevalence was defined as the proportion of inhabitants in the Västra Götaland region given an M54 code during the specific year, stratified by age and gender when appropriate. The Chi-square test was used to calculate any statistically significant differences on the 95% confidence level. Microsoft Excel was used for statistical analyses.

Ethical approval was not needed since only aggregated data were used from the VEGA register, making personal identification impossible.

## Results

In 2014, the total population was 1 632 012, and in 2018, it had increased to 1 709 814. The proportion of women decreased throughout these years from 50.0% to 49.7%. The number of primary health care clinics was 507 in 2014 and 485 in 2018, and the corresponding numbers of outpatient clinics (of all specialties) were 640 and 698, respectively. A consistent figure of 10 inpatient clinics (hospitals) was noted.

**Figure 1.**
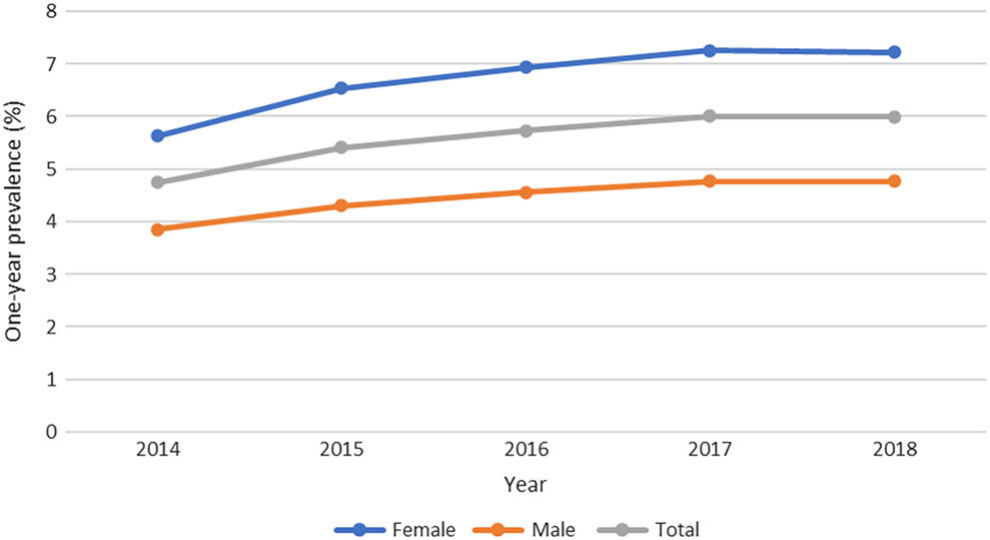
One-year prevalence (%) of M54 in primary health care in Region Västra Götaland

In 2014, the one-year prevalence of M54 in primary health care was 4.8%, and in 2018, it was 6.0%, a 26% increase (*P* < 0.001, CI 25–7%), as shown in Figure 1. In 2018, the total one-year prevalence for all the three levels of health care was 6.6% (Table 1).

**Table 1.** Total one-year prevalence of M54 stratified by level of health care and year in Region Västra Götaland

Clinic	2014	2015	2016	2017	2018
Primary health care	4.8%	5.4%	5.7%	6.0%	6.0%
Outpatient clinic	0.51%	0.50%	0.55%	0.54%	0.52%
Inpatient clinic	0.066%	0.058%	0.052%	0.044%	0.040%

The one-year prevalence of the ICD-10 code M54 within the primary health care was significantly (*P* = 0.001) higher among women in all the studied years. The one-year prevalence of M54 among both women and men increased each year from 2014 to 2017, as did the difference between the sexes. Between 2017 and 2018, the curve flattened. The increase in one-year prevalence from 2014 to 2018 was statistically significant for both women (28%, *P* = 0.001, CI 26–29%) and men (24%, *P* = 0.001, CI 22–26%). In 2018, females had a 51% higher one-year prevalence compared to men (7.2% among women and 4.8% among men, *P* = 0.001, CI 49–52%).

**Figure 2.**
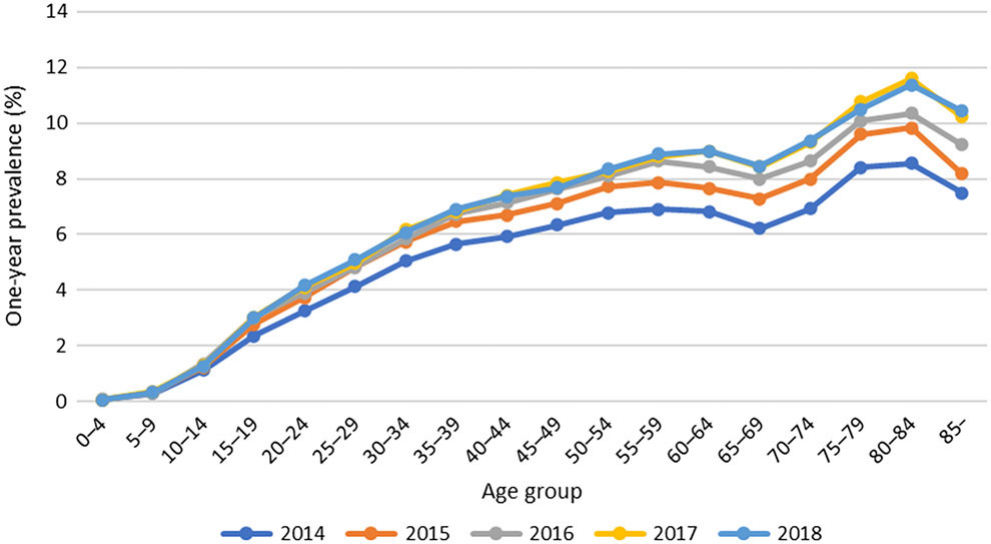
The one-year prevalence (%) of unique individuals given the ICD-10 code M54 in primary health care, stratified by age and year.

The age distribution is displayed in Figure 2. The one-year prevalence of M54 increased with age. The highest figure was seen in the 80–84-years category (11%).

The most common M54 subcodes in 2018 were M54.5, low back pain (2.2%), M54.9, unspecified back pain (2.0%), M54.2, cervicalgia (1.3%), and M54.4, lumbago with sciatica (1.1%). The remaining diagnoses were uncommon, with a one-year prevalence <0.004%. The one-year prevalence was significantly higher (*P* < 0.001) among women in all M54 subcodes.

## Discussion

This study aimed to evaluate the one-year prevalence of diagnosed nonspecific back pain in western Sweden.

The one-year prevalence of M54 in public primary health care in 2018 was 6.0% in Region Västra Götaland and have increased with 26% (4.8% to 6.0%) between 2014 and 2018. The one-year prevalence of this present study was lower than in many previous studies which may be related to differences in study design. This study was register based with a very large number of individuals, and the other studies were self-reporting cohort studies of smaller size (van Tulder *et al.*, 2006; Wenig *et al.*, 2009; Balague *et al.*, 2012). However, even if the methods differ, the results do concur with earlier results concerning the prevalence of visits to the primary health care (6%) in Britain in 2006 due to back pain (Jordan *et al.*, 2010). In accordance with earlier findings, the rather low prevalence in the present study indicate that many patients with back pain do not seek public health care, or use alternative health care instead (Picavet *et al.*, 2008; Ferreira *et al.*, 2010). Other multidisciplinary studies confirm that most people with low back pain can continue to work regardless of their back problems and therefore are probably less likely to seek medical advice (Ehrlich, 2003a; 2003b) or chose to use corporate health care that is not included in this study.

The annual increase in one-year prevalence from 2014 to 2018 may be affected by the inclusion of rehabilitation centers in the VEGA register in 2014 (Bo Palaszewski, 2019). Another contributing factor could be a change in sociodemographic parameters in Västra Götaland because of the recent rise in immigration, mainly from Syria, Afghanistan, and India. Between 2014 and 2018, the net immigration was 76 090 (Sternvik, 2019; Analytics, 2020). According to a governmental analysis in 2015, immigrants were 2.6 times more likely to suffer from back pain compared to the general population even if adjusted for socioeconomic factors (Anna Kjellström, 2019).

The current study found that women are diagnosed with M54 50% more frequently than men (7.2% versus 4.8% in 2018), which is in accordance with a previous review (Hoy *et al.*, 2012). A higher tendency among women to seek medical aid is a possible explanation for these gender differences (Ferreira *et al.*, 2010; Wang *et al.*, 2013). The same conclusion was made in a recent national health care analysis, which found an 85% prevalence for women seeking public health care and a 74% prevalence for men in 2018. The total health care consultation prevalence due to any cause in primary health care was 71%, in outpatient care 47% and in inpatient care 9% (Bo Palaszewski, 2019). In the current study, the diagnosed one-year prevalence of nonspecific back pain among women increased with 28% between 2014 and 2018 (5.65% to 7.22%), compared to a 24% (3.85% versus 4.77%) among men. This might indicate that not only is diagnosed nonspecific back pain more common among women, but the difference in one-year prevalence is also increasing between the sexes.

Not surprisingly and as concluded in previous studies, the one-year prevalence of M54 was the highest among the elderly and uncommon among children (Balague *et al.*, 1999; Hoy *et al.*, 2014; Meucci *et al.*, 2015). The highest one-year prevalence was seen in the 80–84-years category (11%). Degenerative changes are the most common causes of back pain in the elderly (Bono, 2004). The one-year prevalence increased by each age category, but there was a drop in the 65–69-years group. This could be related to the fact that the retirement age in Sweden is 65, and the willingness to seek medical aid is potentially reduced if a sick leave certificate from the doctor is no longer needed.

The most frequently used M54 sub codes in primary health care were those for low back pain, unspecified back pain, cervicalgia, and lumbago with sciatica, which all had a one-year prevalence between 1% and 2.2%. This is to be expected considering that degenerative changes mainly occur in the cervical and lumbar parts of the spine, saving the thoracic spine. Also, the ICD-10 code for unspecified back pain is probably used as a broader diagnosis, ignoring the localization of the pain.

### Strengths and limitations

A major strength of this study is the high reliability of the data since the reporting to the register is mandatory and supervised. The public health care coverage in the VEGA register is 100%. Furthermore, the sample size (frame population) was large. The VEGA register is under governmental control, and the reported diagnoses would therefore be of high data quality. However, there are no publicly available validity data for this register.

Further, M54 is not the only ICD-10 code indicating back pain. Back pain symptoms can be classified elsewhere within the ICD-10 system, for instance as other unspecified diagnoses such as pain (R52.9) or myalgia (M79.1) or specified back pain diagnoses such as disc herniation (M511) or spinal stenosis (M48.0), which are not included in the study. Some patients might be overdiagnosed with a specific back pain diagnosis in combination with an M54 diagnosis, such as disc herniation and lumbago, and are therefore wrongfully included in this study, which aims to study nonspecified back pain.

### Clinical implications

Important clinical findings of this study were an increasing one-year prevalence of nonspecific back pain in public health care and a considerably higher prevalence among women. The nondiagnosed one-year prevalence can be expected to be even higher, as many patients may hesitate to seek medical attention or prefer alternative care. A responsible manner in which to spend public funds is possibly to emphasize female inclusion in both preventive measures as well as in rehabilitation health care programs. Accessibility, structure, and patient-orientated health care are key concepts in the struggle for reducing nonspecific back pain among patients and costs for society. Increased cooperation between publicly funded health care and the alternative health care in future health care programs may be a step in the right direction.

## Conclusion

The one-year prevalence of diagnosed nonspecific back pain was 6% in 2018 and has increased since 2014. Furthermore, women receive this diagnosis considerably more frequently than men. The true one-year prevalence may be higher than was found in this study considering that people seeking help for their nonspecific back pain within the corporate or alternative care, which are not included in the VEGA register. Still, a one-year prevalence number as high as 6% suggests that publicly funded rehabilitation efforts as well as actions focusing on the prevention of nonspecific back pain is money well spent.
